# Development of a wearable belt with integrated sensors for measuring multiple physiological parameters related to heart failure

**DOI:** 10.1038/s41598-022-23680-1

**Published:** 2022-11-24

**Authors:** Sheikh M. A. Iqbal, Imadeldin Mahgoub, E Du, Mary Ann Leavitt, Waseem Asghar

**Affiliations:** 1 Department of Electrical Engineering and Computer Science, Florida Atlantic University, Boca Raton, FL 33431 USA; 2 Asghar-Lab, Micro and Nanotechnology in Medicine, College of Engineering and Computer Science, Boca Raton, FL 33431 USA; 3 Department of Ocean and Mechanical Engineering, Florida Atlantic University, Boca Raton, FL 33431 USA; 4 Christine E. Lynn College of Nursing, Florida Atlantic University, Boca Raton, FL 33431 USA; 5 Department of Biological Sciences (Courtesy Appointment), Florida Atlantic University, Boca Raton, FL 33431 USA

**Keywords:** Cardiology, Health care, Engineering, Biomedical engineering, Electrical and electronic engineering

## Abstract

Heart failure is a chronic disease, the symptoms of which occur due to a lack of cardiac output. It can be better managed with continuous and real time monitoring. Some efforts have been made in the past for the management of heart failure. Most of these efforts were based on a single parameter for example thoracic impedance or heart rate alone. Herein, we report a wearable device that can provide monitoring of multiple physiological parameters related to heart failure. It is based on the sensing of multiple parameters simultaneously including thoracic impedance, heart rate, electrocardiogram and motion activity. These parameters are measured using different sensors which are embedded in a wearable belt for their continuous and real time monitoring. The healthcare wearable device has been tested in different conditions including sitting, standing, laying, and walking. Results demonstrate that the reported wearable device keeps track of the aforementioned parameters in all conditions.

## Introduction

Cardiovascular diseases (CVDs) are a group of heart and blood vessel diseases such as myocardial infraction, commonly known as a heart attack, heart failure, rheumatic heart disease and pulmonary arterial disease^1,2^. According to World Health Organization (WHO), CVDs are the number one cause of death with estimated 17.9 million deaths around the world^1^. Heart failure (HF) is a critical CVD with an estimated 64.34 million cases around the world^3^. HF is a progressive clinical syndrome characterized by a structural abnormality of the heart, in which the heart is unable to pump sufficient blood to meet the body’s requirements. Due to this lack of blood supply, fluid accumulates in the lungs, which impedes oxygenation^4–6^. There are two types of HF: systolic HF with reduced ejection fraction (HFrEF) and diastolic HF with preserved ejection failure (HFpEF). Common causes of HFrEF include cardiomyopathy, heart muscle disease, untreated high blood pressure, faulty heart valves, and coronary artery disease. A common cause of HFpEF is left ventricular hypertrophy (LVH), a condition in which the left ventricle of the heart is thickened and the chamber is unable to sufficiently fill adequate cardiac output^7–9^. According to the Centers for Disease Control and Prevention (CDC), in 2018 there were 379,800 deaths, and 13.4% of total mortality in the US was due to HF^2^. Moreover, according to the American Heart Association, there are currently 6.2 million adults diagnosed with HF in the US, and this number is estimated to increase to 8 million by 2030^10^. Current HF treatment includes guideline-directed medications and surgically implanted devices which can be very costly. According to the CDC, on average, $30.7 billion were expended on the treatment of HF across the US in 2012^2^. This financial burden is due to the downward trajectory of HF which in later stages leads to repeated hospitalization. Due to this poor diagnosis, 17–45% of deaths occur within one year of initial hospitalization and 45–60% of deaths occur within five years^11^.

Continuous and real-time monitoring of HF symptoms can alert patients and providers of patient decompensation. Provider can then intervene with medications to avoid patient hospitalization. Fluid accumulation in the lungs is reflected by a decrease in thoracic impedance. Common symptoms of HF are related to fluid overload and include fatigue, weight gain, and feeling short of breath^7,8^. These symptoms can be monitored for the progression of HF. Currently there are two implantable devices to monitor HF symptoms:, an implantable cardioverter-defibrillator (ICD) and the CardioMEMS™ pulmonary artery monitor^8,12–14^.

An ICD is recommended for patients with HFrEF as they are more likely to have lethal cardiac arrhythmias. An ICD also measures thoracic impedance and can alert providers of the decrease of thoracic impedance, indicating more fluid in the lungs^15^. It is surgically implanted under the skin, and it detects lethal arrhythmias and restores normal heart rhythm with an electric shock. ICDs have an additional function as a pacemaker, to speed up a heart that is too slow^16^. An ICD requires an invasive surgical procedure for the initial implantation and whenever battery needs to be replaced, typically within 3–7 years^17^. There are risks to any surgery and the procedure is also costly. According to the ICD registry, surgical replacement costs approximately $37,000^18^. Moreover, electromagnetic fields can disrupt the ICD performance, and the risk increases with the increased proximity^19–23^. It is important to note that ICDs are only recommended for patients with HFrEF; there are no monitoring devices available for 50% of patients with HFpEF^24^.

CardioMEMS™, is a commercially available diagnostic tool for HF that can alert providers of increased pressure in the lungs. It is a small (15 mm × 3.5 mm × 2 mm) device that is implanted in the pulmonary artery and monitors changes in pulmonary arterial pressure. Increased pulmonary artery pressure is an early indicator of worsening HF ^25–27^. It is costly, approximately $17,750^11^, and not without risk. CardioMEMS™ was approved by Food and Drug Administration in 2014, for both HFrEF and HFpEF, and in its first three years, 5500 devices were implanted in unique patients. However, CardioMEMS™ failed to predict 22 deaths out of 5500 implants, 4 out of which were due to HF^28,29^. Moreover, sensor failure occurred in 46 cases, in which 13 required recalibration, 11 patients were hospitalized and 14 sensors were discarded^28^.

Both currently available HF monitoring systems are not only costly but have significant safety concerns. Moreover, risks of invasive procedures cannot be ignored. Approximately half of patients with HF do not need an ICD and do not qualify for thoracic monitoring it provides. Therefore, there is a critical need for non-invasive solutions for the continuous and real time monitoring of HF progression. Healthcare Wearable Devices (HWDs) can address this need as HWDs are not only cost-effective but are also safe and convenient for the wearer. Moreover, they have been found to be an adequate solution for the continuous and real time monitoring of various biomarkers^30,31^. In addition to ICD and CardioMEMS, remote dielectric sensing (ReDS) from Sensible Medical, also measures lung fluid content but it is also not portable and cannot be used for point of care at all times^32^.

Moreover, VitalPatch by VitalConnect is a portable wearable device that can be used to monitor different vital signs related to cardiovascular diseases^33^. These parameters include heart rate, heart rate variability, respiration rate, body temperature, ECG, posture, and activity fall detection. However, it does not measure thoracic impedance, a significant parameter for the monitoring of HF^33^.

In this paper, we present an HWD that has the potential to monitor physiological parameters that are important for patients with HF. These parameters include thoracic impedance, electrocardiogram (ECG), heart rate, and motion activity detection.

Thoracic impedance is a critical bio-signal for the monitoring of HF progression, having a magnitude in between 60 and 1000 ohms depending upon the subject under consideration and the number of electrodes used for measuring thoracic impedance^34^. As discussed, at the onset of HF, fluid starts to accumulate in the thoracic region, this retention of fluid decreases the impedance in this area. Yu et al. in their study of 33 HF patients observed that before the onset of HF, thoracic impedance starts to decrease^35^. Therefore, this decrease in thoracic impedance is a vital consideration for HF progression^36–38^. Thoracic impedance is evaluated by placing electrodes across the thoracic region, and it measures the resistance to the flow of ions in this area. When the heart is not pumping efficiently, fluid fills the thoracic cavity and facilitates the flow of ions, as fluid is more conductive than air. ^35,39,40^. The increased flow of charges indicates a decrease in the thoracic impedance. Similarly, with the absence of fluid inside the thoracic region, charges face increased resistance to flow from one electrode to another, which indicates an increase in thoracic impedance^41^.

Similarly, ECG is a vital bio-signal for the diagnosis and prognosis of cardiovascular diseases. It is a representation of the flow of electrical signals through the heart^42^. As discussed, one of the symptoms of heart failure is abnormal heart rhythms, known as heart arrhythmias^43^. Heart arrhythmia is an improper beating of the heart in which the ECG is irregular and differentiable from regular sinus rhythm^43^. These heart arrhythmias can be identified using ECG. Traditionally, in the outpatient setting, ECG is measured using a Holter monitor which is not suitable for point of care (POC) use. Moreover, cardiomyopathy causes decreased ejection fraction, in which the percentage of blood pumped with each heartbeat decreases^44^. To compensate for the loss of blood supply, the heart may beat at a higher rate than usual (60–100 beats per minute). This may not be sufficient to provide the cardiac output needed by the body and leads to HF symptoms.

Fatigue is another symptom of HF along with swellings in the legs or edema^7,8^. O’Donnell et al. conducted a study on 13 HF patients and found that severe HF patients were less able to perform physical activity and hence have low activity^45^. Moreover, the discomfort due to heart failure affects sleep patterns^46,47^. These symptoms can be monitored using position sensors which can be used for the better management of heart failure.

This paper will highlight the materials and methods involved in the development of the HWD for the acquisition of the aforementioned parameters along with its preliminary results. Moreover, it will also discuss the challenges and future directions for the use of the discussed HWD for the prediction of HF.

## Materials and methods

**Figure 1:**
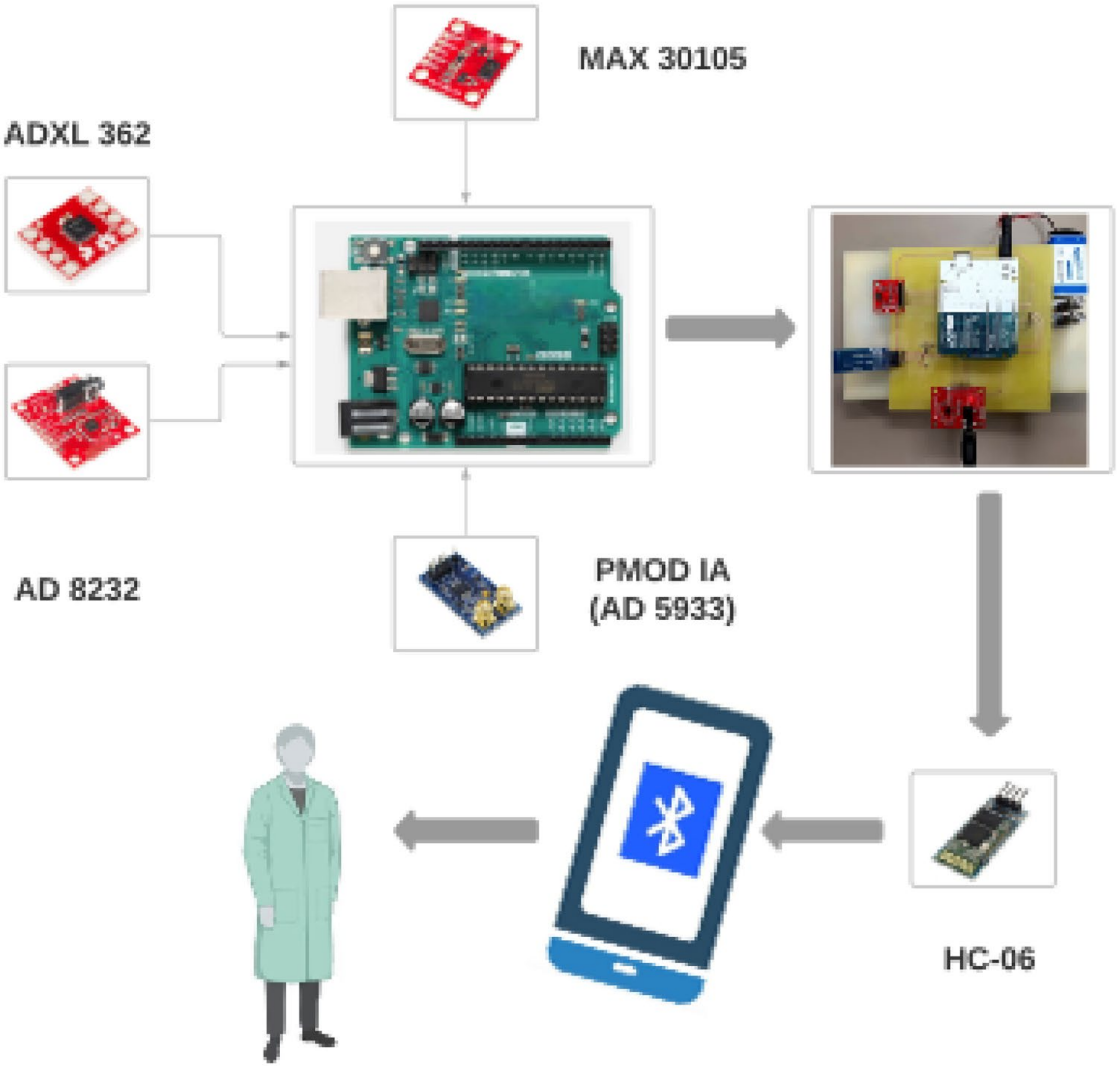
Block diagram of the operation of the HWD for HF prediction showing different sensors used for sensing multiple parameters along with the integrated final module.

The HWD monitors several parameters that are significant for the monitoring of HF^48,49^. As discussed, these parameters include thoracic impedance, ECG, heart rate, and motion activity. Figure 1 presents the block diagram of the proposed HWD. It depicts different sensors being used for monitoring specific parameters. These parameters are then viewed on a smartphone and can be saved to share with the medical provider. The subsequent paragraphs highlight the individual details of these sensors along with their integration towards the development of the final module.

### Materials

As discussed, the presented HWD is based on specific parameters namely thoracic impedance, electrocardiogram (ECG), heart rate, and motion activity detection. These parameters have been found to be significant in determining the symptoms of HF and can potentially be vital in the continuous monitoring of HF symptoms. The system uses different sensors for sensing these parameters. These sensors include a peripheral module interface (PMOD) Impedance Analyzer (IA), for sensing thoracic impedance, AD 8232, for sensing ECG, MAX 30105, for sensing heart rate, and ADXL 362, for motion activity^50–53^. These sensors are then integrated with Arduino Uno as the microcontroller^54^. The subsequent paragraphs discuss these sensors in detail.

**Figure 2:**
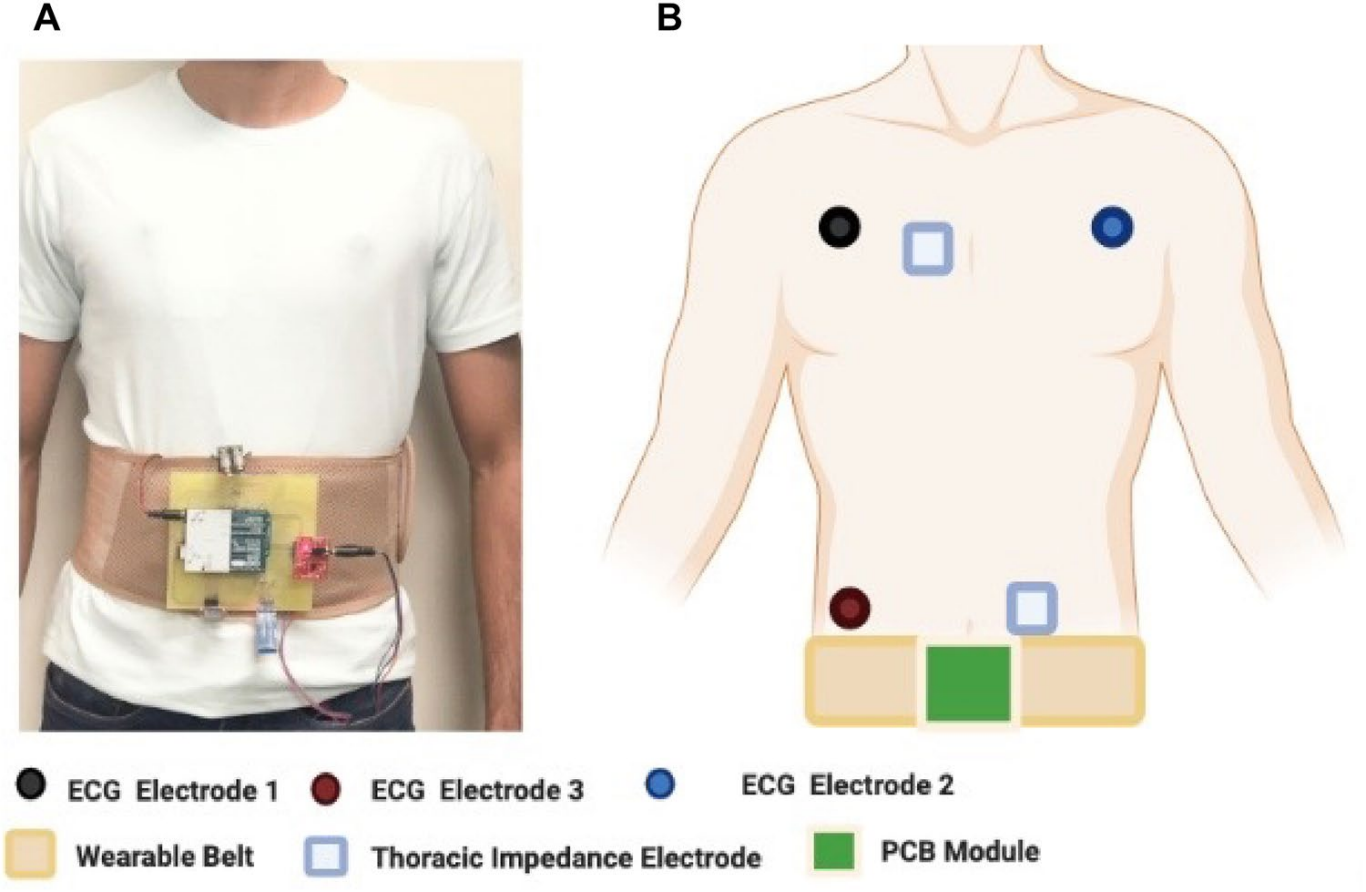
HWD with sensors integrated with the microcontroller (**A**). HWD module on a wearable belt for the continuous and real time monitoring of significant parameters for HF (**B**). Schematic of the HWD showing the placement of the electrodes for the ECG and thoracic impedance.

The PMOD IA has been used to measure thoracic impedance. PMOD IA is a low-cost impedance analyzer for measuring unknown impedances. It is based on AD 5933, a high-precision impedance-converted system, suitable for measuring bioimpedances^50^. AD 5933 has an on-board frequency generator, with a 12 bit, 1MSPS, analog-digital converter^55^. It allows the user-defined frequency sweep, with start and stop frequencies along with the increment in the frequency sweep to excite the external unknown impedance at a known frequency^55–57^. Thoracic impedance is measured against the applied frequency sweep using two electrodes. A known frequency is applied on one electrode and the thoracic impedance response for the applied frequency is captured on the second electrode. For this purpose, electrodes are attached across the thoracic region so as to measure the thoracic impedance in between the electrodes across the thoracic region, as shown in Fig. 2B. Thoracic impedance has a significant response for frequencies in between 10 and 100 kHz and therefore, PMOD IA in this HWD has been programmed to obtain impedances for this frequency range^34^. The real and imaginary parts of the response are stored on AD 5933 built-in registers^57^.

Several integrated circuits, with comparable performances, are available that can be used in HWDs for ECG measurement at POC. AD 8232 is one such integrated circuit that measures ECG using a single lead^51^. AD 8232 has been used in the proposed HWD to record ECG. As the performance of the ECG largely depends on the placement of electrodes, therefore the electrode position was finalized after trying different placements such that they are connected in Einthoven’s triangle^58^. Figure 2B shows the final placement of ECG and thoracic impedance electrodes.

The MAX 30105 sensor can be used to measure heart rate. MAX 30105 is a powerful and flexible sensor for detecting heartbeat^52^. Its sensing is based on the absorption of light by the oxygenated blood with every heartbeat^52^. The optics of the MAX 30105 are explained in the supplementary information Sect. 1.1.

ADXL 362 is a widely used position sensor for motion activity, position analysis, and monitoring sleep patterns^53^. It is a 3-axis MEMS accelerometer that measures the change in all three axes (x, y, z)^53^. It measures both the accelerations: static acceleration, such as tilt and dynamic acceleration, such as that due to shock or motion^53^. The aforementioned sensors are integrated for the development of an HWD that can be worn at all times, as shown in the Fig. 2.

### Methods

The aforementioned sensors are used to read significant parameters of HF and are integrated using Arduino Uno. Arduino Uno is a low-power microcontroller that allows 5–12 V of voltage and consumes a maximum of 42 mA current^54^. It allows both the inter-integrated circuit (I^2^C) and the serial peripheral interface (SPI) modes of communication. The subsequent paragraphs highlight the details of the final module with the integration of these sensors.

Thoracic impedance (AD5933) and heart rate (MAX 30105) sensors use I^2^C mode of communication whereas position (ADXL 362) and ECG (AD8232) sensors use SPI mode of communications. Arduino Uno provides four pins for I^2^C communication: two for each serial data line (SDA) and serial communication line (SCL). Therefore, one pair of SDA and SCL pins is used for reading thoracic impedance data and one for reading heart rate data. Similarly, digital pins of the Arduino Uno allow SPI mode of communication and are used for reading data from position and ECG sensors.

An Arduino sketch with built-in libraries for the aforementioned sensors was written. These libraries allow to send and receive instructions from the general-purpose input/output (GPIO) pins of the Arduino. All these instructions were run in a loop to ensure continuous transfer of data from the Arduino pins. After the sketch is burnt on the Arduino, the thoracic impedance sensor is calibrated with a known resistor. For this purpose, an 82 Ω resistor has been used, because it lies within the human thoracic impedance range. Once calibrated, the thoracic impedance electrodes are inserted in the place of the calibrated resistor and PMOD IA sense thoracic impedance with reference to the calibrated resistor. All other sensors do not need any calibration and can be used as it is. Moreover, the ECG recording is sampled at around 204 Hz for accurate results. The integrated module is tested under different conditions for example, sitting, standing, walking, and laying. A study protocol for this purpose, defined in Table 1, has been implemented on a health subject. In all these conditions an experiment has been designed to test the change in thoracic impedance. As thoracic impedance is the reflection of the resistance to the flow of charges inside the thoracic region therefore the inflow and outflow of air inside the thorax change its impedance. The healthy subject under consideration performs aggressive inhaling and exhaling to observe the change in thoracic impedance in thorax region and PMOD IA was used to record this change in thoracic impedance^59,60^. However, all other sensors sequentially record the respective parameters, as shown in Table 1.

**Table 1:** The study protocol being followed in each condition.

Condition	Experiment	Approximate duration (min)
Sitting	Heart rate Thoracic impedance Normal breathing Aggressive inhaling Aggressive exhaling Motion activity detection/position analysis ECG	3.5
Standing	Heart rate Thoracic impedance Normal breathing Aggressive inhaling Aggressive exhaling Position analysis ECG	3.5
Walking	Heart rate Thoracic impedance Normal breathing Aggressive inhaling Aggressive exhaling Motion activity detection/position analysis ECG	3.5
Laying	Heart rate Thoracic impedance Normal breathing Aggressive inhaling Aggressive exhaling Motion activity detection/position analysis ECG	3.5

The study was approved by the Institutional Review Board committee of Florida Atlantic University for the aforementioned experiment and all experiments have been performed in accordance with the relevant guidelines and regulations. Moreover, informed consent has also been obtained from all participants, of the study.

### Final Module

**Figure 3:**
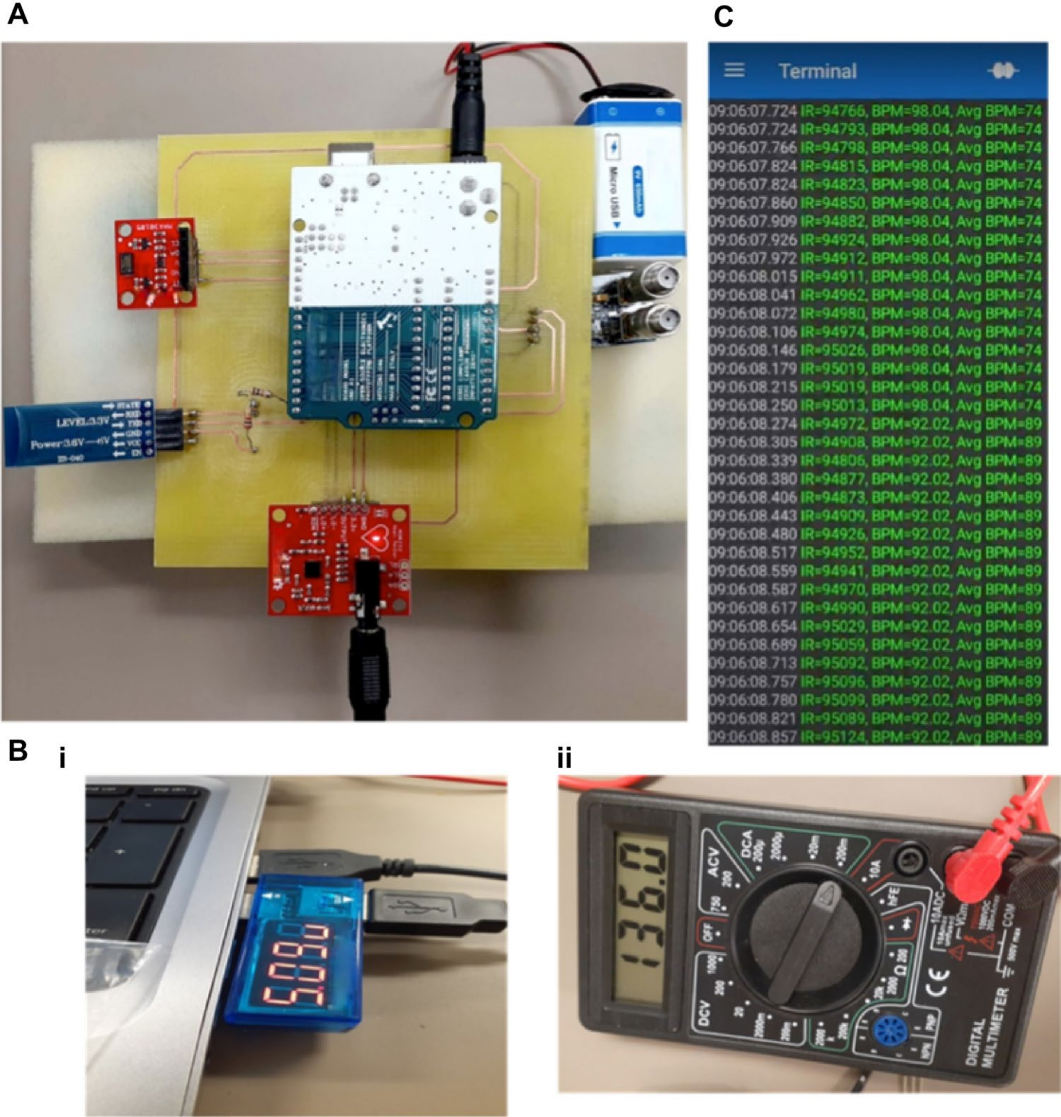
(**A**). HWD for heart failure prediction (**B**). Power specifications of the HWD i. Input voltage of the HWD ii. Current required by the HWD (** C**). Heart Rate in beats per mins being displayed on smartphone using Bluetooth application.

The sensors are integrated on a single microcontroller to monitor multiple parameters with Arduino Uno as a microcontroller. The final module is shown in Fig. 3A. The module is lightweight with 120.1 × 125 mm in dimension making it convenient to be worn by the user. The maximum power consumption of the module is 0.6 W with 136 mA current and 5 V, as shown in Fig. 3B. This power consumption allows the module to use a small compact-size battery, suitable for acquiring data over a long period of time. A Li-ion battery of 650 mAh capacity has been used for this purpose. The battery allows around 4.7 h of uninterrupted acquisition of data before fully discharging and takes only a few hours to recharge. This uninterrupted data is essential for the continuous and real-time monitoring of HF.

The results from the HWD can be transmitted to user’s mobile phone using Bluetooth module, as shown in the Fig. 3C^61^. For this purpose, HC-06 has been used as a Bluetooth module and results are visualized on a serial Bluetooth terminal (SBT) application^62,63^. SBT application is a free application to receive data from Arduino’s serial monitor using Bluetooth. SBT can be used to receive data from the HWD for real time visualization of the results and data can be saved for later use. Moreover, it can also be used to log data to share it with the medical practitioner for future use. Video of the real time sharing of data using Bluetooth module can be found in supplementary Sect. 1.2.

### Informed consent

Informed consent was obtained from all subjects involved in the study.

## Results

As discussed, the HWD was tested under different conditions to evaluate its efficacy as a wearable device. For this purpose, results for all the sensors were obtained in sitting, standing, walking, and laying conditions. In each condition, results were obtained for all sensors sequentially where for thoracic impedance values for normal breathing as well as for aggressive inhaling and exhaling were obtained. This is demonstrated in the video provided in the supplementary Sect. 1.2. Results were obtained for the healthy male subject of age 25 and weight 129 lb with no prior cardiovascular progression medical history.

### Thoracic impedance

**Figure 4:**
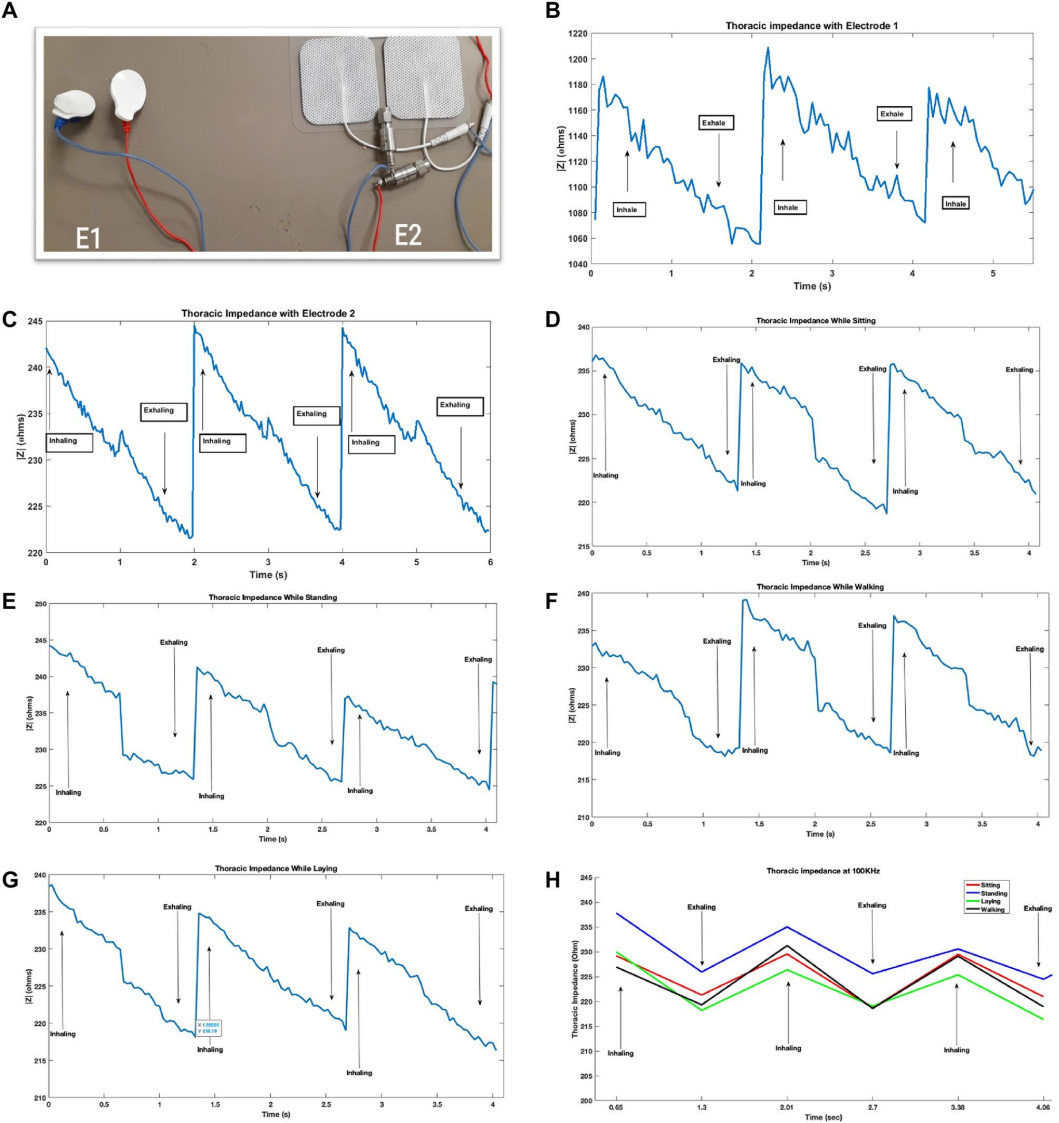
Thoracic impedance using PMOD IA (**A**). Two electrodes with different areas: Electrode E1 with area of 452 mm^2^and electrode E2 with area of 2580 mm^2^ (**B**). Magnitude of thoracic impedance with Electrode E1 (**C**). Magnitude of thoracic impedance with electrode E2. |${\left|\mathrm{Z}\right|}_{\mathrm{E}1}>{\left|\mathrm{Z}\right|}_{\mathrm{E}2}$ Z|_E1_ >|Z|_E2_ (**D**). Thoracic impedance in sitting position (**E**). thoracic impedance in standing position (**F**). thoracic impedance in laying position (**G**). Thoracic impedance while walking (**H**). Thoracic impedance in different conditions at 100 kHz.

The results of thoracic impedance for a male subject are shown in Fig. 4. To analyze in a wider spectrum, we have performed a frequency sweep from 80–100 kHz for different real-time conditions and have plotted all the real values of impedance because biological cells respond to all these frequencies. The sensor gives real and imaginary values of thoracic impedance. Real values of impedance have been considered and plotted because it is the real value of the thoracic impedance that varies with the fluid accumulation and is significantly correlated with heart failure^64,65^. We have shown this by plotting thoracic impedance values for both inhaling and exhaling.

It can be seen from Fig. 4 that the thoracic impedance increases while inhaling and decreases while exhaling. The significant increase in thoracic impedance while aggressive inhaling is due to the presence of increased air inside the thorax region and as air is less conductive therefore the total impedance of the thorax region increases^59,66^. Similarly, the significant decrease in thoracic impedance while aggressive exhaling is due to the absence of air and is well in accordance with the expected outcomes of the thoracic impedance^59,67,68^.

The results from PMOD IA are also cross-validated by using two electrodes of different areas, as shown in Fig. 4A. According to Piuzzi et al. the thoracic impedance decreases with the increased area of the electrode being used to evaluate it. ^34^. Figure 4B,C shows the thoracic impedance obtained using electrodes with two different areas. The same experiment of thoracic impedance while inhalation and exhalation are performed on the same subject. Impedance from both electrodes retain the expected trend. However, Electrode 1, with area of 452 mm^2^, has a thoracic impedance greater than the magnitude of thoracic impedance obtained using Electrode 2, having an area of 2580 mm^2^^69^. This is in accordance with the findings of the aforementioned study and validates the results of the PMOD IA^34^. As the results of electrode 2 are found to be more robust and noise free therefore further results were obtained using electrode 2. Electrode 2 is a self-adhering surface electrode by Auvon having low impedance and longer adhesiveness^70^.

Thoracic impedance was evaluated for different conditions while sitting, standing, laying, and walking for real-time and continuous monitoring. Results for these conditions are shown in Fig. 4D–G, respectively. It can be seen that under all conditions the PMOD IA senses the change in thoracic impedance with a magnitude ranging from 218-250Ω for the subject under consideration. As expected, thoracic impedance increases while inhaling and decreases while exhaling in all conditions. Moreover, it can be seen as well that there are not many variations in the thoracic impedance while in motion for example while walking. This shows that PMOD IA has successfully mitigated the motion artifacts to have clean signals. In consideration to the referenced work, we have also included a plot that only shows the values of thoracic impedance at 100 kHz and it can be seen that for inhaling it increases, and for exhaling it decreases, as shown in Fig. 4H.

### Electrocardiogram (ECG)

**Figure 5:**
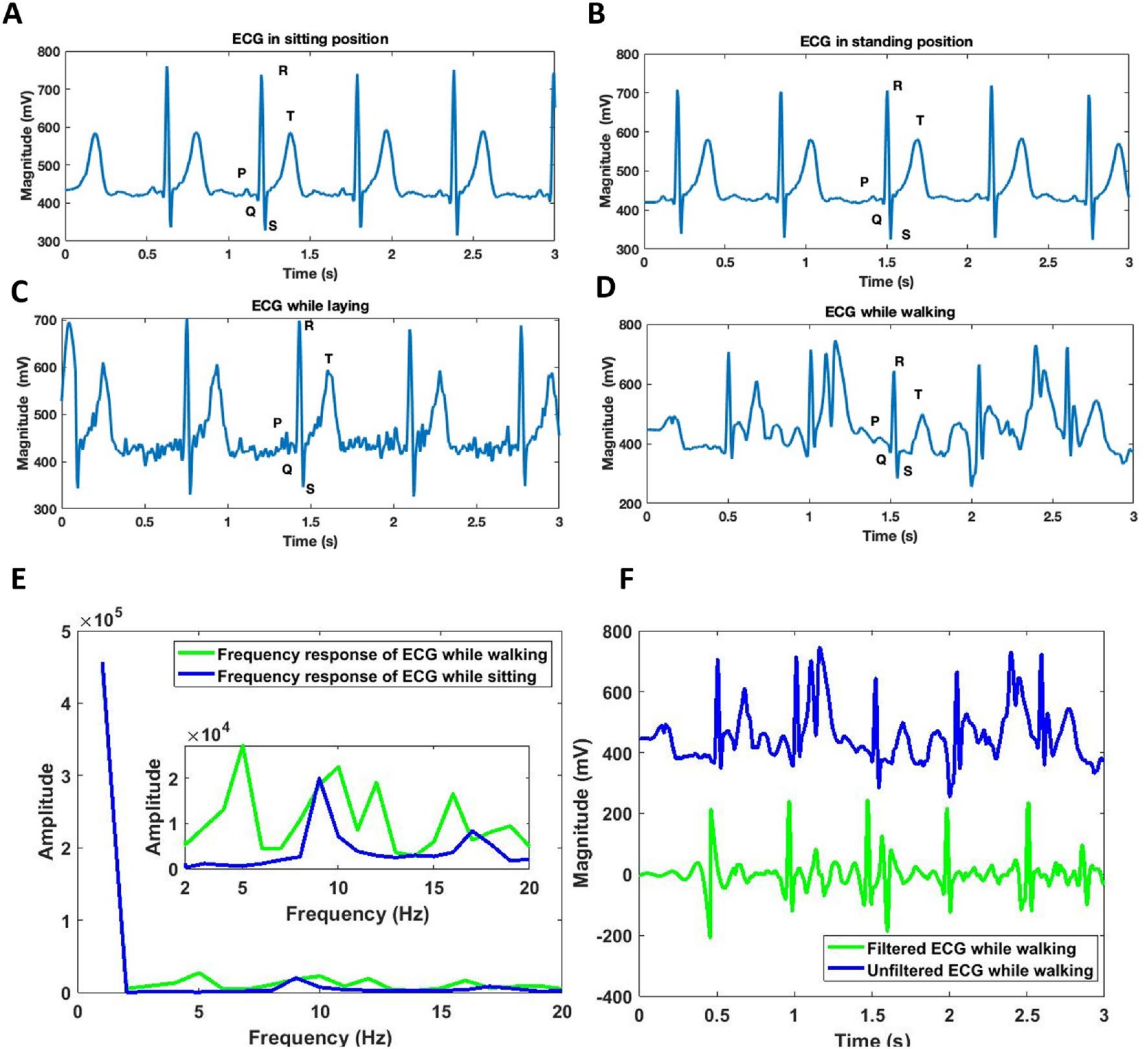
An ECG of a healthy subject with a clean and visible PQRST complex under different conditions (**A**). ECG while sitting (**B**). ECG while standing (** C**). ECG while laying (** D**). ECG while walking (**E**). Frequency response of ECG while sitting and while walking (**F**). ECG while walking before (blue) and after the high pass filter (green).

As discussed, AD 8232 IC has been used for recording ECG in the developed HWD. Results for the ECG under different conditions were obtained and are shown in Fig. 5A–D. It can be seen that a visible PQRST complex is obtained with ECG clean enough to be used for prognosis and diagnosis purposes. A clean ECG has been obtained under stable conditions for example, while sitting, standing, and laying however, the ECG while walking is distorted. The ECG while walking is distorted due to the expected inclusion of motion artifact due to movement. However, in walking, the QRS complex of the ECG is still visible and can be utilized for prognosis purposes without the need for further signal processing, as shown in Fig. 5D.

As the frequency response of motion artifact (0.01–10 Hz) coincides with the frequency response of the ECG (0.5–100 Hz), it is difficult to filter motion artifacts from the ECG, as there remains a tradeoff in the removal of motion artifacts and retaining the low-frequency signals of ECG^71^. However, improvements in the ECG while walking can be made by removing the low-frequency components of the motion artifacts using a high pass filter. Figure 5e shows the frequency response of the ECG while walking and of a clean ECG while sitting. It can be seen that additional frequency components are present in the ECG, while walking, from 4–6 Hz. These components can be removed using a high pass filter with a stop frequency of 4 Hz and pass frequency of 7 Hz. For this purpose, a finite impulse response (FIR) high pass filter of type equiripple has been used and specifications of which can be found in the supplementary information Sect. 1.3. ECG under walking condition after passing through the aforementioned high pass filter is shown in Fig. 5f. It can be seen that the filtered ECG does not have the baseline wander in comparison to the unfiltered ECG while walking and therefore is centered around 0.

### Heart rate

The MAX 30,105 has been used to measure heart rate. It measures the heart rate based on the absorption of infrared (IR) rays. The sensor is placed next to the body on the belt, so as to absorb the IR rays under all conditions. It has been observed that for the subject under consideration the average heart rate remains in the expected range of 60–100 bpm at all times.

**Figure 6:**
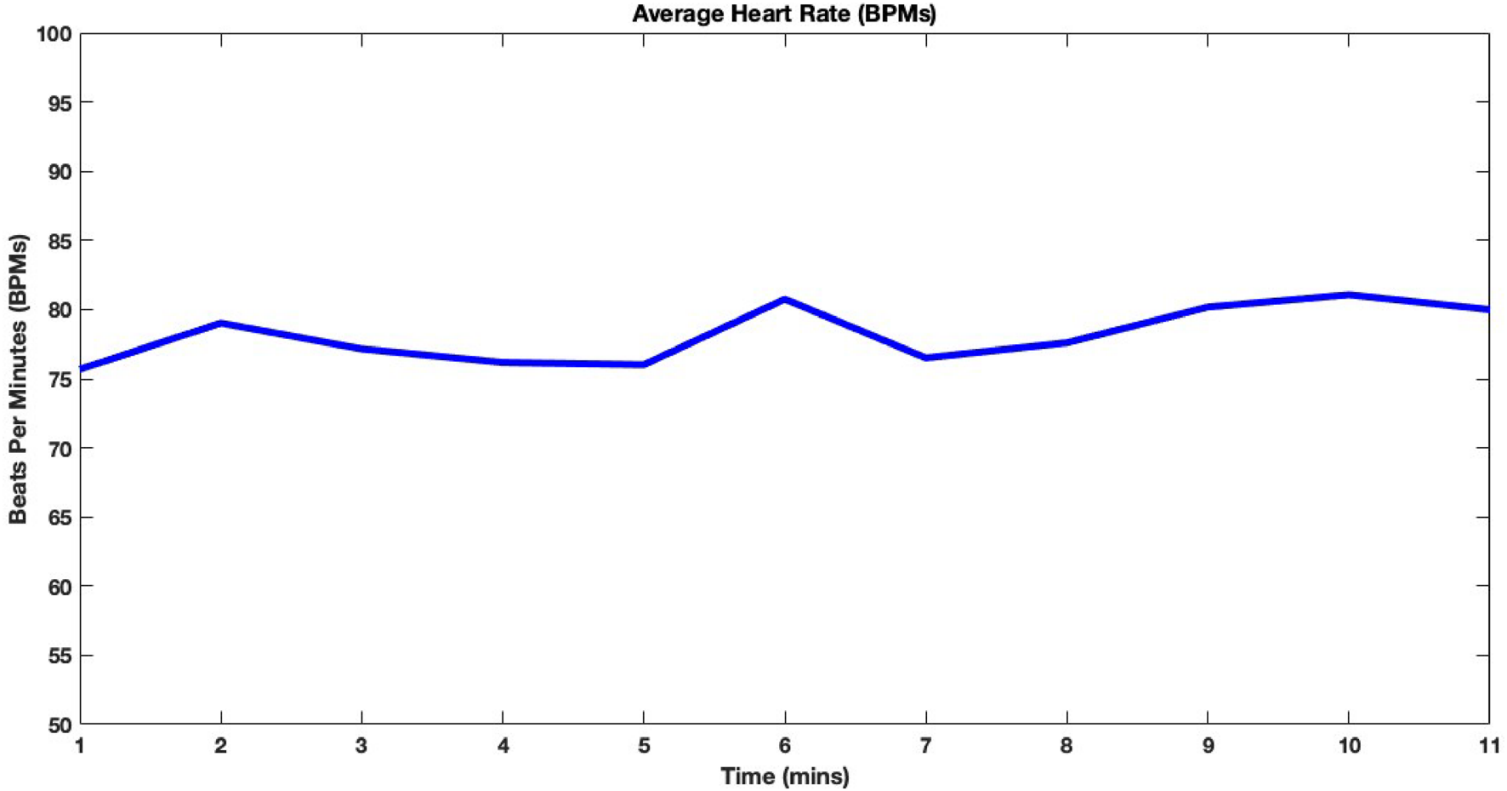
Real time heart rate values in bpm for subject under consideration.

Figure 6 shows the results of the heart rate for a healthy male subject in beats per-minute (bpm). MAX30105 gives average heart rate values in bpms. Heart rate fluctuates due to unequal pressure on the sensor therefore we have filtered the values in the expected heart rate range (60–100 bpms) and took the per minute average of them. Complete heart rate data is also provided in the supplementary information Sect. 1.5.

### Motion activity detection

**Figure 7:**
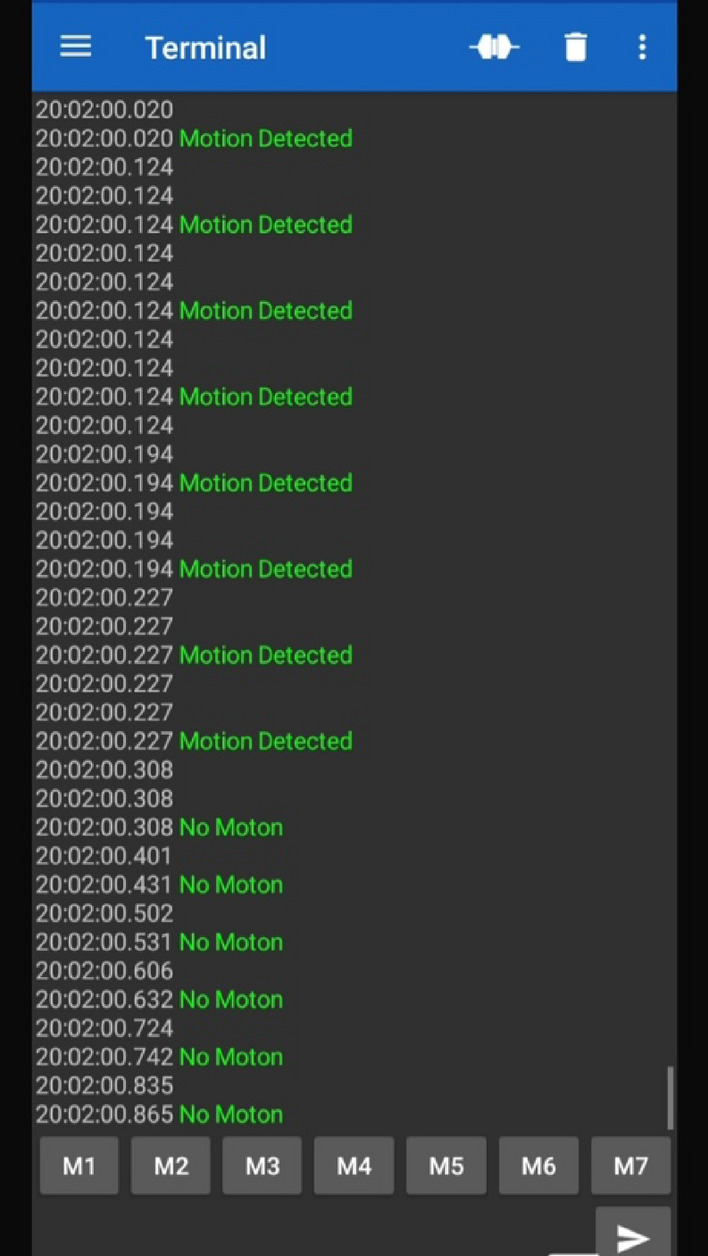
Motion Detection using ADXL 362 being visualized on a Bluetooth application.

As discussed, ADXL 362 has been used to keep track of the motion of the subject, an essential parameter towards the diagnosis of heart failure. The ADXL 362 detects the motion whenever the motion of the subject is higher than the set threshold and for a set amount of time. As discussed, ADXL also gives values of acceleration that can be used to differentiate different positions of the subject. Results of the motion activity detection are shown in Fig. 7 and position analysis of the subject under consideration for different conditions is shown in Table.2. It can be seen that the sensor correctly detects the change in axis with change in position. At different positions the values of the axes are different for example, while laying the y-axis has negative values in comparison to the sitting, standing and walking positions where y-axis is positive. Moreover, the change in magnitude of y-axis from sitting to standing position also highlights the accuracy of the position sensor.

**Table 2:** Results from position sensor under different positions.

X axis	Y axis	Z axis	Position
XVALUE = 247	YVALUE = 832	ZVALUE = −1400	Sitting
XVALUE = 275	YVALUE = 921	ZVALUE = −1172	Standing
XVALUE = 440	YVALUE = 934	ZVALUE = −1113	Walking
XVALUE = 200	YVALUE = -305	ZVALUE = −2020	Laying

## Discussion

A prototype of the HWD for the continuous and real-time monitoring of vital biosignals has been developed that can be used for the monitoring of HF. The HWD reads parameters such as thoracic impedance, ECG, heart rate, and motion activity detection. The HWD has been tested under different conditions and the corresponding results for different human states have been obtained. It can be seen from the results that all sensors kept track of the changes in different conditions.  The position sensor correctly highlights the change in position in different conditions, as shown in Table 2, and can also be used to identify different states of the wearer. Moreover, the heart rate sensor also always keeps track of the heart rate, as shown in Fig. 6. The HWD also correctly highlights the minute changes in the thoracic impedance as well, as shown in Fig. 4. It was observed that the thoracic impedance is not notably affected with the change in position and for a given subject the average thoracic impedance remains same as should be expected. It has been observed that the ECG sensor, like most ECG monitors, is very sensitive to motion and often incorporates motion artifact during movement, particularly while walking, as shown in Fig. 5D. However, even while walking the ECG does retain its QRS complex along with R-peaks, as can be seen in Fig. 5D, which are important indicators for LVH. The obtained ECG while walking can be used to diagnose LVH using modified Cornell criteria to indicate the increased amplitude of R peak in augmented vector left (aVL) ECG where LVH, as discussed, is a significant cause of heart failure^72–74^. Moreover, in the proposed HWD the thoracic impedance sensor is calibrated manually but once calibrated it does not require further calibration given the module is used uninterruptedly. All the sensors are integrated into a belt module that can easily be worn for a long period of time without affecting the patient’s daily activities. The bluetooth module has also been used to enable automated sensor data transfer, without any user input, to the terminal for further analysis.

According to Gyllesten et al. thoracic impedance is a strong predictor of HF in comparison to short term weight gain^40,75^. Thoracic impedance alone has the prognostic ability to predict HF much earlier than the weight monitoring^35^. On a study conducted by Yu et al. on 33 patients, 25 incidents of hospitalization occurred in 10 patients and in all patients the daily measured thoracic impedance was found to be lower than their respective baseline thoracic impedance for an average of 18 ± 10.3 days before hospitalization^35^. Similarly, Vollmann et al. conducted a study to monitor the change in thoracic impedance in 373 HF patients using an implant device^38^. The device is programmed with an algorithm that generates an alert whenever the thoracic impedance is lower than the reference thoracic impedance. It has been observed that for 53 clinical HF events, the algorithm detects HF deterioration with a sensitivity of 62%. Moreover, in another study ECG was monitored for 6664 patients, with 244 HF events observed. The ECG of all these events exhibited higher resting heart rate, left ventricular hypotrophy, extended QRS duration, abnormal ST/T wave and abnormal QRS-T^76^. Similarly, in another study, it has been observed that the ECG does have high sensitivity, around 89%, for HF but it has low specificity for HF, around 56% ^77^. Therefore, ECG alone can be associated with other cardiovascular diseases and can lead to incorrect prediction.

Therefore, a multiparameter approach has been adopted and a device has been developed to monitor multiple parameters. It is expected that the proposed HWD will have higher predictive values for HF with increased specificity and high sensitivity. With the aforementioned results, it is planned to test the module over a set of diverse subjects in the near future, and an algorithm can be developed to predict heart failure over the test set. If an anomaly is observed, a notification can be sent to the wearer for further interventions. This notification can also be sent to the concerned medical provider.

## Conclusion

A HWD has been proposed for monitoring parameters that are important for the better management of HF. The HWD uses multiple parameters that can provide essential information about HF. These parameters are thoracic impedance, heart rate, ECG, and motion activity detection. The HWD consumes power of the order of 0.6 W and therefore can be powered with a lightweight battery. The battery used has a capacity of 650 mAh that allows the module to continuously monitor the data for at least 4.7 h and can be fully recharged in a few hours. The preliminary results obtained from the HWD under varied conditions are encouraging and can be used to develop an algorithm, in the next stage of the project, for predicting heart failure. The proposed HWD is lightweight and is 120.1 × 125 mm in dimensions that can conveniently be worn on the waist.

## Supplementary Information

Supplementary Information.

## Data Availability

The datasets generated during and/or analyzed during the current study are available from the corresponding author on reasonable request. They are also provided in the supplementary information document.

## Code availability

An open-access link to the code, used for the acquisition of these signals can be found in the supplementary Sect. 1.6.
