# Supplementary material for: Development of a wearable belt with integrated sensors for measuring multiple physiological parameters related to heart failure
Source: Sci Rep. 2022 Nov 24;12:20264. doi: 10.1038/s41598-022-23680-1 (PMC9691694; doi:10.1038/s41598-022-23680-1)
Supplement: Supplementary file 1 — Supplementary Information. [file 41598_2022_23680_MOESM1_ESM.docx]

**Development of a wearable belt with integrated sensors for measuring multiple physiological parameters related to heart failure**

Sheikh MA Iqbal^1,2^, Imadeldin Mahgoub^1^, E Du^3^, Mary Ann Leavitt^4^ and Waseem Asghar^1,2,5,*^

^1^ *Department of Electrical Engineering and Computer Science, Florida Atlantic University, Boca Raton, FL 33431*

^2^ *Asghar-Lab, Micro and Nanotechnology in Medicine, College of Engineering and Computer Science, Boca Raton, FL 33431, USA*

^3^ *Department of Ocean and Mechanical Engineering, Florida Atlantic University, Boca Raton, FL 33431*

^4^ *Christine E. Lynn College of Nursing, Florida Atlantic University, Boca Raton, FL 33431*

^5^ *Department of Biological Sciences (Courtesy appointment), Florida Atlantic University, Boca Raton, FL 33431, USA*

* Correspondence: wasghar@fau.edu

**Supplementary Information**

- 1. **Optics of MAX 30105**

Image below shows the optics behind the MAX 30105. MAX 30105 contains LEDs and photon detector where the photon detector detects the light reflections from the particle under consideration in this case the oxygenated blood. As hemoglobin reflects IR light, the MAX 30105 filters the heartbeat from the reflection of IR using the photon detector.


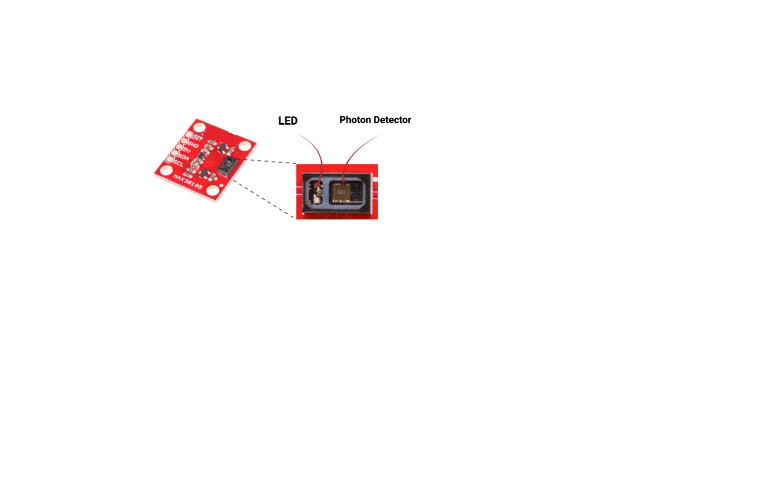


**Fig.1** MAX 30105 Optics containing LEDs and photon detector.

- 1. **Video of the Experiment with data being shared using Bluetooth module:**

Video of the experiment can be found in this link:

<https://youtu.be/8IxTNS8Dt8s>

- 1. **High Pass filter specifications and MATLAB code**

Fstop = 4;

Fpass = 7;

Apass = 0.01;

Astop = 80;

filtSpecs = fdesign.highpass(Fstop,Fpass,Astop,Apass,Fs);

bpFIR = design(filtSpecs,'equiripple','SystemObject',true);

ecg_filtered=filter(bpFIR.Numerator,1,ecg_noisy);


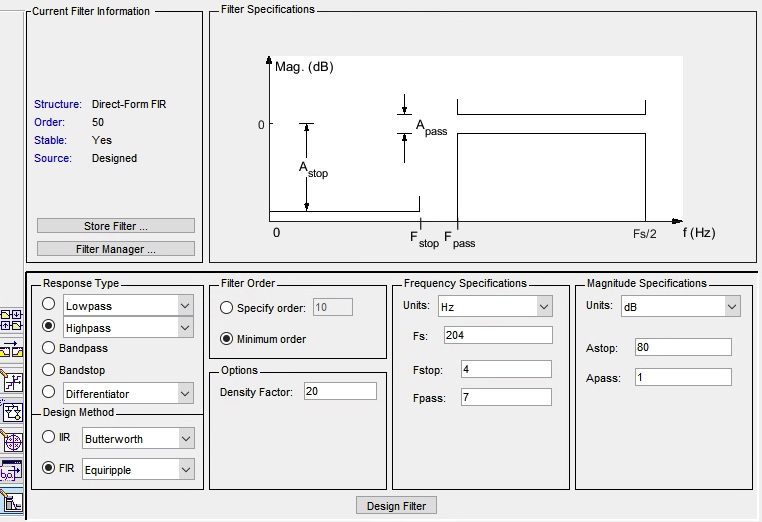


**Fig.2** High pass filter design using MATLAB.

**1.4. Arduino Code**

Arduino Integrated design environment (IDE) is divided into two main functions of type void: void setup () and void loop (). Setup () is used for initializing the pins of Arduino with required sensors/ components and loop is used for reading and writing from those pins. Below block diagram describes the structure of code used for continuously recording the discussed parameters.

**Fig.3** Structure of Arduino code.

**1.5. Heart Rate Data file link:**

<https://github.com/ShkhAsher/Development-of-a-wearable-belt-with-integrated-sensors-Code.git>

**1.6. Code Availability Link:**

<https://github.com/ShkhAsher/Development-of-a-wearable-belt-with-integrated-sensors-Code.git>
